# Socially segregated, sympatric sperm whale clans in the Atlantic Ocean

**DOI:** 10.1098/rsos.160061

**Published:** 2016-06-08

**Authors:** Shane Gero, Anne Bøttcher, Hal Whitehead, Peter Teglberg Madsen

**Affiliations:** 1 Department of Zoophysiology, Institute of Bioscience, Aarhus University, Aarhus, Denmark; 2 Department of Biology, Dalhousie University, Halifax, Canada

**Keywords:** culture, clan, communication, social structure, dialect, geographical variation

## Abstract

Sperm whales (*Physeter macrocephalus*) are unusual in that there is good evidence for sympatric populations with distinct culturally determined behaviour, including potential acoustic markers of the population division. In the Pacific, socially segregated, vocal clans with distinct dialects coexist; by contrast, geographical variation in vocal repertoire in the Atlantic has been attributed to drift. We examine networks of acoustic repertoire similarity and social interactions for 11 social units in the Eastern Caribbean. We find the presence of two socially segregated, sympatric vocal clans whose dialects differ significantly both in terms of categorical coda types produced by each clan (Mantel test between clans: matrix correlation = 0.256; *p* ≤ 0.001) and when using classification-free similarity which ignores defined types (Mantel test between clans: matrix correlation = 0.180; *p* ≤ 0.001). The more common of the two clans makes a characteristic 1 + 1 + 3 coda, while the other less often sighted clan makes predominantly regular codas. Units were only observed associating with other units within their vocal clan. This study demonstrates that sympatric vocal clans do exist in the Atlantic, that they define a higher order level of social organization as they do in the Pacific, and suggests that cultural identity at the clan level is probably important in this species worldwide.

## 1. Introduction

The formation of social boundaries based on culture was critically important to the evolution of humans. Cultural boundaries are often the limits of cooperative and altruistic exchanges; among humans, language helped to identify these social boundaries in order to solve the dilemma of with whom to cooperate [1–4]. These variations in vocal repertoire marked cultural structures in human society which in turn affected reproduction, survival and selection, and paved the way for large-scale cooperative societies [5,6]

Among mammals, variation in vocal repertoire between sympatric and parapatric groups which could potentially interbreed has generally been referred to as *dialects*, while differences between populations separated by long distances which do not interbreed has been referred to as *geographical variation* [7]. Geographical variation of vocal repertoires is widespread across mammalian taxa, including small terrestrial mammals [8], bats [9], monkeys (e.g. [10]), apes (e.g. [11]) and marine mammals (e.g. [12,13]). This type of variation is often the result of long-term isolation between populations and may be driven by genetic distinctiveness. Alternatively, if vocal learning does occur and cultural segregation mirrors geographical separation, vocal repertoires can diverge over time through spatial or demographic factors with copying errors and random cultural drift creating variation between isolated communities [14–16]. Once either of these occurs, the behavioural differences between the two communities can itself lead to genetic distinctiveness and reproductive isolation [17].

Sympatric dialects, however, appear to be quite rare and are thought to be the result of selection to advertise the distinction between groups. Along with high levels of philopatry and the consequent learning biases [18,19], vocal learning is thought to be the primary mechanism in the evolution and maintenance of dialects among local, interacting groups. While common in songbirds, vocal learning is rare among mammals. The cetaceans are an interesting exception with advanced vocal imitation and learning abilities [20–22].

The sperm whale (*Physeter macrocephalus*) provides an interesting case study for the evolution of culturally transmitted dialects as the pattern of variation in vocal repertoires differs between ocean basins. In both the Pacific and Atlantic Oceans, social units of female sperm whales produce repertoires of ‘codas’ [23,24], which are stereotyped patterns of three or more broadband clicks [25]. In the Eastern Tropical Pacific, Rendell & Whitehead [23] described over 70 different coda types and used differing coda production repertoires to define distinct ‘vocal clans’ in which dozens of social units shared a similar coda repertoire. Units exclusively associate with units within their vocal clan, even though clan habitats are sympatric, suggesting that the dialects, and/or possibly other signals, function as markers of a shared cultural heritage and may act as barriers to cooperative and altruistic behaviour [5,6,23]. Recent work shows that acoustic variation among clans can neither easily be explained by genetic variation [26], nor is it the product of stochastic processes such as cultural drift [19]. Modelling reveals that biases in vocal learning of coda types, specifically conformity (preferentially learning the most common coda types, [27]) and homophily (learning from behaviourally similar individuals due to social modularity, [28]), are required to generate the cultural segregation observed in the Pacific [19].

The current understanding of vocal variation among sperm whales in the North Atlantic Ocean is quite different. Coda repertoires in the Atlantic vary geographically and there is a significant negative correlation between repertoire similarity and spatial distance between populations [29]. This finding suggests that cultural drift could have played a role in the evolution of divergent repertoires. Gero *et al.* [24] found no evidence of sympatric dialects in the Eastern Caribbean. However, one particular coda type (‘1 + 1 + 3’) was found to be produced with very high levels of stereotypy across the entire community, to have only ever been recorded in the Caribbean and to have remained the dominant coda type in the region for at least the last 30 years. The 1 + 1 + 3 coda's stability over this timeline and ubiquity across a population divided into disparate social units who range widely provides a rare example of cultural transmission maintaining high levels of conformity of a behaviour. This finding would indicate that biased vocal learning is also occuring in the Atlantic and that the conformity in the production of this coda type might function as a marker of clan membership in the Eastern Caribbean [24]. Nonetheless, given the lack of sympatric dialects, it begs the question of what might be the function of this level of conformity—why signal clan membership if there is only one clan present, and is social assorting not necessary or possible?

A potential solution to this conundrum was suggested by the patterns observed in sympatric clans in the Pacific. Units from different clans used the same waters, but tended to appear in temporal waves, associating with other units of their own clan [30]. Perhaps, some of the rarely identified Eastern Caribbean units, which were generally encountered apart from the common units, were members of one or more separate clans. During the longitudinal research project off the island nation of Dominica, we frequently encountered nine social units (units A, D, F, J, N, R, T, U and V from [31]; mean sightings days from 2005 to 2015: 89, range: 42–184) and these have been the subjects of intense social and acoustic analysis [24,31–40]. Here, we compare the vocal repertoires for two rarely seen units (units P and K in [31]) which have been identified only on 16 and 7 days, respectively, across the 10-year study, testing the hypothesis that they are from a separate clan or clans. We demonstrate that the two rare units produce a shared repertoire which is distinct from that of the nine common units and provide the first evidence of socially segregated, sympatric vocal clans in the Atlantic Ocean.

## 2. Material and methods

### 2.1. Field methods

Social units of female and immature sperm whales were located and followed in an area that covered approximately 2000 km^2^ along the entire western coast of the island of Dominica (15.30° N, 61.40° W). Unit membership was designated based on previous photo-identification analysis in which whales were unit members if they associated in sequential years and in which association was defined as identified within 2 h of each other [31]. The multi-year requirement places emphasis on the long-term bonds among unit members and will also be conservative by including only those members for which long-term spatio-temporal coordination is evident [31]. Research was conducted in the winters of 2005 through 2015 for a total of 3660 h with whales encountered on 402 days across 472 days of effort at sea (see electronic electronic supplementary material, table S1).

Acoustic recordings were made of sperm whale codas in two contexts: (i) at the onset of deep foraging dives and (ii) when whales were socializing in groups at the surface. Recordings were made with various systems that all had flat frequency responses across ranges of at least 2–20 kHz and sampling rates of 44.1 kHz or higher (see the electronic supplementary material for details). The variation in the recording systems did not affect our ability to record clear signals for both the coda and echolocation clicks produced by sperm whales, and as a result, the temporal patterning of clicks used in this analysis.

### 2.2. Analyses

#### 2.2.1. Measuring and testing of similarity between repertoires

To define the temporal structure of the codas recorded, we measured absolute inter-click intervals (ICI, the time between the onset of one click and the onset of the next click in the coda; see the electronic supplementary material for methodological details). We calculated the absolute ICI measures for recordings from units P and K and used the existing dataset of absolute ICI measurements from the nine common units collected by Gero *et al.* [24] using identical methods. To quantify similarity between unit repertoires, we used two different approaches: one categorical and one classification-free continuous measure as in previous work [24,41].

In the categorical approach, two codas were given a similarity of 1 if they were assigned to the same type and were given a similarity of 0 if they were assigned to different types. We assigned codas of similar click length to a categorical type using a hierarchical clustering algorithm called OPTICS [42] run on the absolute ICI measures for all 11 units combined. We used a *ξ*-value of 0.04 for all coda lengths (which defines a 4% drop in point density as the criterion for defining a new cluster) as this best defined the clusters visually evident in plots of the first two components of a principal components analysis (PCA) run on the same data. OPTICS allows for points that are outliers or located in sparse areas between dense clusters to be labelled as noise, rather than being forced into defined clusters. All codas which were not classified into clusters and labelled as noise by OPTICS were omitted from the categorical analysis but retained in the classification-free, continuous measure of similarity described below. We used the OPTICSxi module in the ELKI framework (http://elki.dbs.ifi.lmu.de/, [43]) to run these analyses.

Coda types were given names based on the mean temporal click pattern for all codas included in that cluster, following previous nomenclature [23,24,39,44]. For example, a ‘5R’ coda is one in which five clicks are regularly spaced, while a ‘1 + 1 + 3’ coda sounds like ‘click-[PAUSE]-click-[PAUSE]-click-click-click’ with longer gaps between the first two clicks followed by three clicks in quick succession. We refer to coda types that make up greater than 10% of a unit's coda production as ‘predominant’ coda types.

For the classification-free, continuous measure, the multivariate similarity of two codas with the same number of clicks was measured as a Euclidean distance between the ICI vectors using absolute ICI measures for all 11 units combined using methods as in previous work [41]. Similarities were calculated using custom-written routines in MATLAB 7.12 (The Mathworks, Inc., MA, USA).

Matrix correlations and Mantel tests with 10 000 permutations [45,46] were used to examine repertoire variation between units. We tested whether pairwise similarities between repertoires were higher for repertoires of the same unit on different days (same unit, different day—SUDD) compared with those from different units (different unit, different days—DUDD). Each day's recordings were treated as independent in an attempt to account for any autocorrelation in coda production within a recording day. To do so, we tested the matrix of pairwise similarities of each day's recordings against a 0/1 matrix with 1 coding for SUDD and 0 coding for DUDD. If units produced distinct repertoires, then the expectation is a significantly positive correlation between these matrices. This Mantel procedure was repeated with a 0/1 matrix of the same clan/different clan in which the expected alternative hypothesis is a significant positive correlation between matrices if clan repertoires differ. All matrix correlations and Mantel tests were carried out using SOCPROG 2.5 [47] in MATLAB 7.12 (The Mathworks, Inc., MA, USA).

We then used similarity matrices between repertoires to construct average-linkage clustering dendrograms and measured their robustness using 1000 bootstrap replicates. The cophenetic correlation coefficient (CCC) was also calculated to indicate how well the dendrogram represented the data. A CCC of over 0.8 is considered a ‘good’ representation of the associations [48].

To visualize social and acoustic similarity of the units among clans, we plotted a social network of associations among individual members of the 11 social units overlaid with an acoustic network among units. The edges of the social network were weighted using a half-weight index (HWI) of association [49]. We defined an association between whales when they were photographically identified within a cluster at the surface (40 m chain rule) within a 2 h sampling interval using photo-identification data from 2005 to 2015, as in previous work [32]. The acoustic network was built based on coda repertoire similarity and weighted by the measures of multivariate similarity between unit repertoires, as above. The social network was plotted using Gephi 0.8.2beta (https://www.gephi.org) and a Force Atlas 2 layout algorithm (http://bit.ly/1kmVfe5). This is a force-directed layout in which nodes repel each other, while edges attract nodes they connect. A buffer was given to prevent overlap of different units in order to allow for overlay of larger nodes and the acoustic network.

## 3. Results

We identified 388 codas from 21 recordings on 3 days in 2 years for unit P and 426 codas from 19 recordings on two different days in 1 year for unit K. These were added to the 4116 codas assigned to the nine common units during previous analyses [24], for a total of 4930 codas in the complete dataset for all 11 units. A total of 324 codas (6.6%) were omitted as noise by OPTICS. The remaining 4606 codas were classified into 22 different coda types (see the electronic supplementary material, figure S1 for a plot illustrating rhythm of all coda types). Codas of five clicks in length made up 70.9% of all codas recorded. The unit repertoires were dominated by three prevalent coda types which each made up greater than 10% of all codas recorded: ‘1 + 1 + 3’ (32.7%), ‘5R1’ (21.9%) and ‘5R3’ (12.4%). Mantel tests confirmed that recordings of the same unit on different days are more similar than recordings of different units on different days (table 1). While discovery curves are not asymptotic, they do suggest that repertoires of all units were adequately sampled given that all units have more than approximately 250 codas (electronic supplementary material, figure S2).

**Table 1.** Mean repertoire similarities within and between units recorded off Dominica using two similarity methods. Multivariate similarity based on Euclidean distances between absolute inter-click intervals and basal similarity of 0.001 s. Categorical similarity using OPTICS classification [42] into coda types. Mantel tests have a null hypothesis that repertoire similarity between recordings of the same unit/clan on different days (within) is the same as that between recordings of different units/clans on different days (between). Rejection of the null indicates significantly different repertoires between units or clans.

	multivariate similarity				categorical similarity			
social level	within	between	matrix correlation	*p*-values	within	between	matrix correlation	*p*-values
11 units	0.010	0.006	0.189	<0.001	0.267	0.155	0.190	<0.001
2 clans	0.007	0.002	0.180	<0.001	0.189	0.016	0.256	<0.001

**Figure 1.**
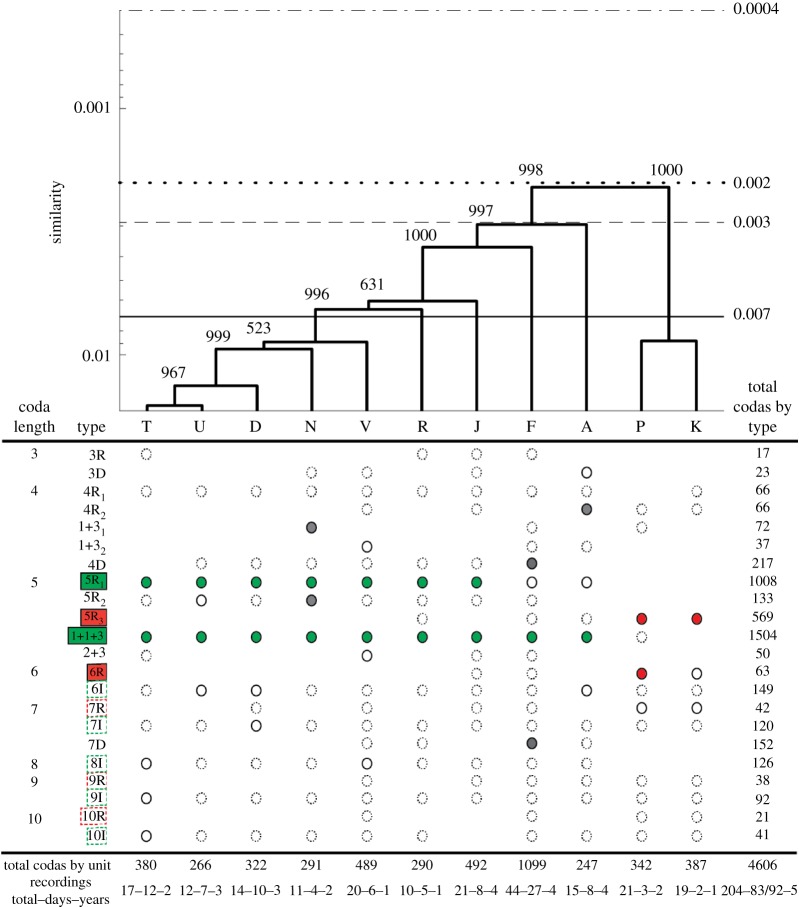
Coda repertoires of units of sperm whales recorded off Dominica compared using Euclidean distances of absolute inter-click intervals (ICIs) with a basal similarity of 0.001 seconds (top) and OPTICS [42] categorical classification into types (bottom). Numbers next to branches of the dendrogram indicate the number of the 1000 bootstrap replicates in which that branch was reproduced. This is a good representation as the dendrogram has a cophenetic correlation coefficient of 0.9784. Horizontal rules indicate the mean within (solid) and between (dotted) clan similarities from the Caribbean and the mean within (dashed lined) and between (dashed-dotted) clan similarities from the Pacific. Note that absolute similarity values may be less meaningful when compared across populations than relative ones within a given area. Letters denote units. Circles in the classification table denote the presence of the coda type in the units' repertoire. Dashed circles indicate types that made up less than 5% of the production, while filled circles made up greater than 10% of a unit's repertoire (predominant codas). Green fill denotes predominant codas in the Eastern Caribbean vocal clan, while red fill marks predominant codas in the new clan (units P and K). Coda type nomenclature: ‘R’ indicates a coda with regular ICIs, ‘+’ indicates a longer gaps between clicks, ‘D’ indicates decreasing ICIs throughout the coda, ‘I’ indicates increasing ICIs throughout the coda and the sequential numbering of the same name (e.g. 5R1, 5R2, 5R3) indicates coda types with the same rhythm but of increasing duration. Numbers below each column are the total number of codas recorded from each unit, as well as the total number of recordings, recording days and years per unit. On 8 different days, recordings were made of multiple units hence that day was counted once as a recording day for each of the units in the unit totals and, therefore, there is a difference in the two totals for days (unique calendar days/unit days).

The 11 social units were divided into two evident clades by a single bifurcation at the root of the hierarchical clustering dendrogram (figure 1). Units P and K were distinguished from the nine common units by distinctly clustering in 100% of the 1000 bootstrap replicates giving confidence that the division is not dependent on sampling. Units P and K share a distinct repertoire from that of the nine commonly observed units, as indicated by both categorical and continuous measures (figure 1 and table 1). Vocal repertoires between the two clades differed significantly based on Mantel tests (table 1). Therefore, using the same justification as in the Pacific [23], we will divide the social units observed off Dominica into the common EC1 vocal clan and the rare EC2 vocal clan.

The most obvious distinction between the two vocal clans is the production of different predominant coda types. In the case of the EC1 clan, the 5R1 and 1 + 1 + 3 codas are predominant types, while the EC2 clan never produced the 5R1 and only unit P produced the 1 + 1 + 3 very infrequently (figure 1; electronic supplementary material, table S2). Instead, the 5R3 coda dominates the repertoires of the EC2 clan and is only produced infrequently by three of the nine common units and in the case of unit P, the 6R coda is also a prevalent type (only two common units infrequently produce the 6R coda). These differences produce the strong division in the structure of the dendrogram in figure 1. Furthermore, the EC2 clan differ consistently from the more common EC1 clan in their production of the non-predominant types. The EC2 clan produced other ‘regular’ class coda types with more clicks (7R, 9R and 10R highlighted in the red dashed boxes in figure 1) which are long in duration (approx. 1.5 s; rhythms plotted in electronic supplementary material, figure S1) and have constant ICIs throughout the coda, while the EC1 clan units produced ‘increasing’ class codas (6I, 7I, 8I, 9I and 10I highlighted in green dashed boxes in figure 1) which are short in duration (less than 0.5 s; rhythms plotted in electronic supplementary material, figure S1) and have increasing ICIs throughout the coda. These distinctions between the two vocal clans are highlighted using plots of the first two principal components by coda type and unit in the electronic supplementary material, figure S3.

**Figure 2.**
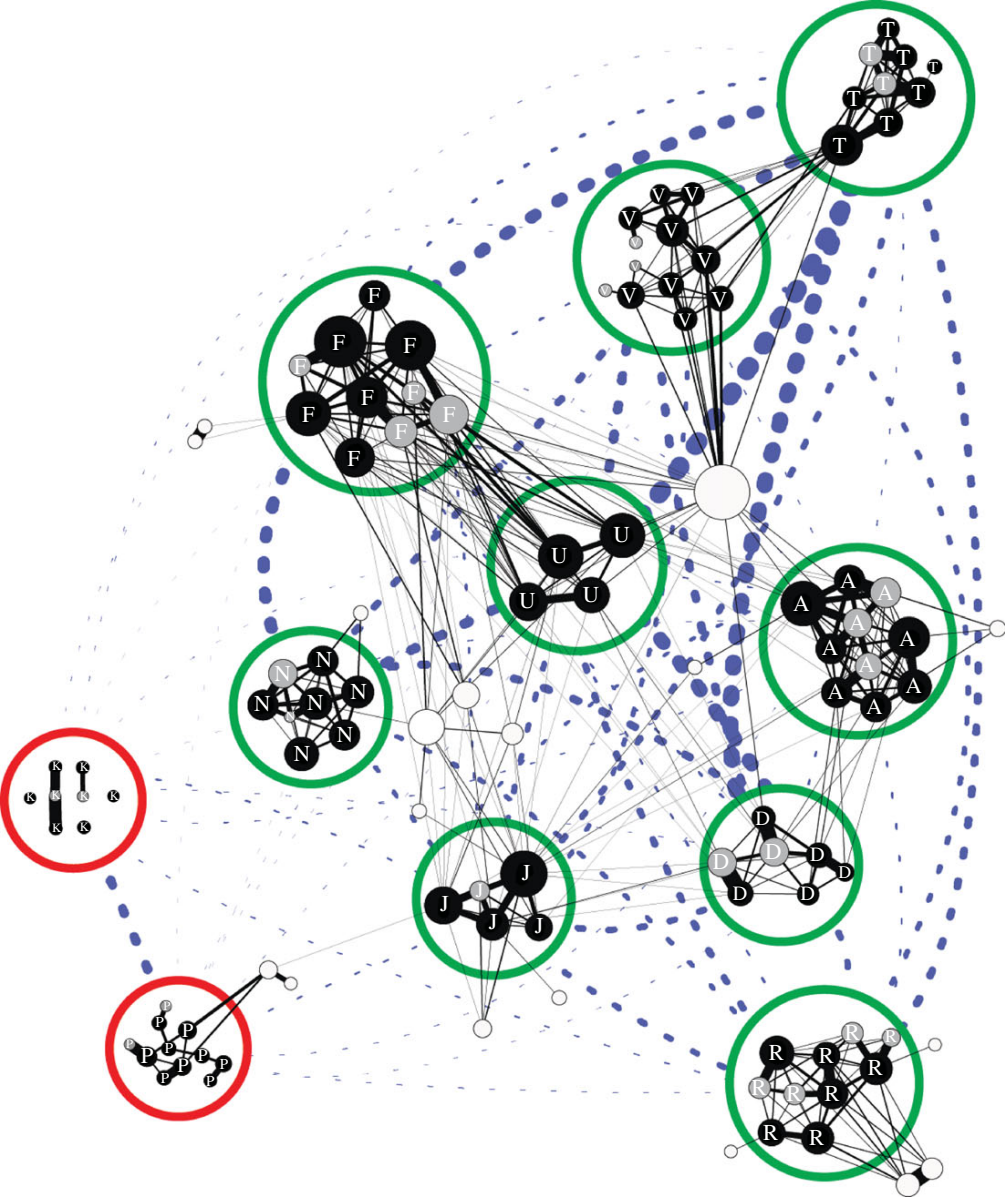
Multidimensional network depicting patterns of social interactions and acoustic similarity across three levels of sperm whale social structure in the Eastern Caribbean. Individuals (small nodes) within units (large nodes) and within vocal clans (colour of large nodes). In the social network, individuals are connected by social relationships (black solid lines) weighted based on the half-weight index of associations based on photo-identification. Individual nodes are sized relative to their measure of degree (number of connected individuals) and coloured based on class (black, adult females; grey, dependent calves; white, mature male escorts). The only social connection between clans is a mature male escort who associated with both unit P and unit J and there are no direct associations between females in differing clans. In the overlapped acoustic network, units (large nodes) are connected by coda repertoire similarity (dashed blue lines) weighted based multivariate similarity and coloured based on clan (green, Eastern Caribbean Clan; red, two rare units). Note that the weighting of the edges for acoustic similarity and social association differ and the relative thickness of lines between social and acoustic networks are not related.

The social network in figure 2 demonstrates the social segregation between clans as there are many associations among units within the common EC1 vocal clan (green circles) but a complete absence between vocal clans. The only social connection between unit P and the EC1 clan is via a mature male who was observed consorting with two units in different clans. The overlaid acoustic network highlights the dissimilarity between clan repertoires and reflects the division in the dendrogram from figure 1.

## 4. Discussion

This study supports the hypothesis generated by the social and cultural patterns observed in the Pacific: in the North Atlantic, as in the Pacific, there are sympatric clans of social units of female sperm whales with distinctive repertoires, who tend to appear in a study area in temporal waves. This finding refutes the contention that repertoire diversity in the Atlantic Ocean is a simple geographical variation caused by cultural drift (genetic drift is an unlikely cause of the dialects as nuclear genes show little geographical structure, [50]). Modelling by Cantor *et al.* [19] suggests that two factors are needed in order to partition Pacific sperm whale units into vocally marked cultural groups, conformism and homophily. Previous research of the Eastern Caribbean sperm whale dialects and sociality has found evidence of both conformism to prevalent coda types [24] and high social modularity [32]. Taken together, these findings demonstrate that social boundaries are structured along cultural lines and support the contention that cultural identity is important in this species in both the North Atlantic and the Pacific.

While our discovery of sympatric clans in the waters off Dominica narrows the gap between our understanding of the socio-cultural systems of sperm whales in the North Atlantic and the Eastern Tropical Pacific, there are still substantial contrasts. In the Pacific, sympatric coda repertoires are evident from each analysis of the vocalizations of female sperm whales based on samples spanning more than a few months in a particular study area ([23,51]; M. Cantor 2015, unpublished data). However, it took many years of data and a determination to analyse the repertoires of the rarer social units before sympatric clans were identified in the Caribbean. While samples for the EC2 clan units are small in terms of number of days on which recordings were made, it is unlikely that additional sampling would yield different results given the clear division between clans maintained by bootstrapping, as well as the different prevalent codas types between the clans which are produced in the majority of one clan's recordings and virtually none in that of the other clan (see the electronic supplementary material for details on sampling and discovery curves). Nonetheless, the image that appears is of social units from one clan, EC1, predominating in our study area and neighbouring waters, with members of the EC2 clan, whose core range is elsewhere, making occasional incursions. The relatively high resighting rates and residency times of the EC1 member units off islands in the Eastern Caribbean [31], taken with the fact that no whales in this study have been identified outside of the Caribbean Sea despite longstanding effort in the neighbouring waters of the Bahamas, Gulf of Mexico, and the western North Atlantic [31,35], suggest that EC1 member units' home ranges, as well as their wider movements, are probably smaller than those described for the Pacific [23,52,53]. Thus, as suggested in the work of Antunes [29], Atlantic clans may overlap in range much less than in the Pacific; nonetheless, we have demonstrated that there are areas in which they are sympatric. Furthermore, the repertoire similarity between clans in the Pacific is an order of magnitude lower than the one between the EC1 and EC2 clans (mean between clan similarity this study: 0.002, s.e. = 0.0001 versus 0.0004, s.e < 0.0001 between sympatric clans in the Pacific; Pacific values re-calculated using Euclidean distance and absolute ICIs, as in this study, from data reported in [22]). While it is likely that the absolute similarity values are less meaningful when compared across populations based on distinct datasets, than relative ones within a given area's dataset, it appears as though the repertoires of sympatric Pacific clans are more distinct than those of sympatric clans in the Caribbean. Variation in the degree of sympatry probably affects the variation in repertoire similarity by altering the selective forces acting to distinguish repertoires among clans. Certainly, this is the case among humans, in which the correlation between symbolic markers and behaviour is the strongest when cultures are close in space [6]; and birds, in which songs in sympatry are more divergent than those in allopatry (e.g. [54]). With increased habitat overlap, one would expect more divergent coda repertoires, should they function in cultural recognition and identity. Our results then follow from the social identity hypothesis proposed by Rendell & Whitehead [23] for the function of coda dialects among Pacific clans. Furthermore, this study answers the question of why there exists such high levels of conformity in the production of the 1 + 1 + 3 coda by members of several disparate social units raised by Gero *et al.* [24]. With the presence of two sympatric clans, highly conserved, clan-characteristic codas (such as the 1 + 1 + 3 coda in the case of the EC1 clan) may function as a marker of cultural identity.

Whitehead & Gero [55] proposed that the Eastern Caribbean, some of the most urban habitat for this species in the North Atlantic Ocean, is an attractive sink (or ecological trap, [56,57]) such that immigration from surrounding waters probably explains the conflicting results of high levels of mortality in this community but an increasing population trend based on mark–recapture data. This may support the scenario outlined in the previous paragraph, whereby the increasing incursions by members of a peripheral EC2 clan would impact mark–recapture estimates, but it would not affect the measures of mortality, recruitment and fecundity which are predominantly based on individual-level long-term study of the most common units in the EC1 clan. Alternatively, units P and K may be recent immigrants from a clan elsewhere in the Atlantic where resources are not as abundant. Electronic supplementary material, figure S4 contrasts the Caribbean coda types with those of neighbouring waters, including the Gulf of Mexico, Azores and the Sargasso Sea from the results of Antunes [29]. It suggests that the vocal repertoire of the EC2 vocal clan may share some similarities with the repertoires in the Sargasso Sea and the Azores in which long ‘Regular’ class codas also predominate. Further quantitative analysis will be required to conclusively match the EC2 clan's repertoire to a specific region in the North Atlantic or determine whether it is a distinct repertoire from those which have been previously documented.

As in the Pacific, units in the Eastern Caribbean seem to be socially segregated based on vocal clan. Identifiable vocal dialects create population structure in this species as they do among killer whales (*Orcinus orca*, [17]), birds (summarized in [58]) and humans. Among human cultural groups, a socially learned vocal marker, language, helps to solve the dilemma of with whom to cooperate by reliably identifying those who share similar behaviours [1,2]. When in-group favouritism of this kind occurs, it can dramatically decrease within-group variation and accordingly increase between-group heterogeneity of other behaviours [59]. Previous work has demonstrated this to be the case among the sympatric clans in the Pacific, which differ in their movement patterns, habitat use and foraging success [30], as well as diet [60], reproductive success [61] and social behaviour [62]. Killer whale ecotypes also show differences across a myriad of behaviours including social interactions, diet and foraging behaviour, and movement patterns and diving behaviour (summarized in [17]). This study opens the door to studies on the cultural content of the two clans in the Caribbean.

## 5. Conclusion

Overall, it would appear that sperm whales in the Eastern Caribbean live in a much more individualized society than their counterparts in the Eastern Tropical Pacific. Social relationships between unit members are dynamic [33], long-term relationships between units may be based on social preferences through direct personal knowledge [32] and, at the highest level, sympatric cultures are segregated based on coda dialect. By contrast, the society in the Pacific appears to be more structured at the cultural level, in an ‘Us versus Them’ fashion, such that units associate with other units which share vocal dialect but do not appear to show strong preferences at lower levels of social organization. This may be the result of a society devastated by whaling, such that the social structure in the Eastern Caribbean reflects what might have existed prior to whaling [53,63]. These differences could also be a response to differing environments in which broad recognition of clan membership is important in the Pacific to facilitate frequent group formation in a high predator risk environment in which individual units may range more widely and are not able to form preferred associations between units [52]. Nonetheless, this study demonstrates that sympatric vocal clans do exist in the Atlantic, that they define a higher order level of social organization as they do in the Pacific, and suggests that cultural identity at the clan level is probably important in this species worldwide.

## Supplementary Material

Gero et al - FULL ESM. This document contains description of supplementary methodological details, as well as supporting figures and tables.

## Data accessibility

The ICI and association data used in this study are available from Dryad Digital Repository (http://dx.doi.org/10.5061/dryad.53g73).

## References

[1] Nettle D, Dunbar RJM 1997 Social markers and the evolution of reciprocal exchange. Curr. Anthropol. 38, 93–99. (doi:10.1086/204588)

[2] Nettle D 1999 Language variation and the evolution of societies. In The evolution of culture (eds Dunbar RIM, Knight C, Power C), pp. 214–227. Piscataway, NJ: Rutgers University Press.

[3] Van den Berghe PL 1981 The ethnic phenomenon. New York, NY: Elsevier.

[4] Barth F 1969 Introduction. In Ethnic groups and boundaries: the social organization of cultural difference (ed. Barth F), pp. 9–38. Boston, MA: Little, Brown and Company.

[5] Boyd R, Richerson PJ 1987 The evolution of ethnic markers. Cult. Anthropol. 2, 65–79. (doi:10.1525/can.1987.2.1.02a00070)

[6] McElreath R, Boyd R, Richerson PJ 2003 Shared norms and the evolution of ethnic markers. Curr. Anthropol. 44, 122–129. (doi:10.1086/345689)

[7] Conner DA 1982 Dialects vs. geographic variation in mammalian vocalizations. Anim. Behav. 30, 297–298 (doi:10.1016/S0003-3472(82)80269-8)

[8] Kershenbaum A, Ilany A, Blaustein L, Geffen E 2012 Syntactic structure and geographical dialects in the songs of male rock hyraxes. Proc. R. Soc. B 279, 2974–2981. (doi:10.1098/rspb.2012.0322) 10.1098/rspb.2012.0322 PMC3385477 22513862

[9] Wilkinson GS 2003 Social and vocal complexity in bats. In Animal social complexity: intelligence, culture, and individualized societies (eds De Waal FBM, Tyack P), pp. 322–341. Cambridge, MA: Harvard University Press.

[10] Maeda T, Masataka N 1987 Locale-specific vocal behaviour of the tamarin (*Saguinus I. labiatus*). Ethology 75, 25–30. (doi:10.1111/j.1439-0310.1987.tb00639.x)

[11] Wich SA et al. 2012 Call cultures in orangutans? PLoS ONE 7, e36180 (doi:10.1371/journal.pone.0036180) 22586464 10.1371/journal.pone.0036180 PMC3346723

[12] Thomas JA, Stirling I 1983 Geographic variation in the underwater vocalizations of Weddell seals (*Leptonychotes weddelli*) from Palmer Peninsula and McMurdo Sound, Antarctica. Can. J. Zool. 61, 2203–2212. (doi:10.1139/z83-291)

[13] Payne RS, Guinee LN 1983 Humpback whale, *Megaptera novaeangliae*, songs as indicators of ‘stocks’. In Communication and behaviour of whales (ed. Payne RS), pp. 333–358. Boulder, CO: Westview Press.

[14] Podos J, Warren PS 2007 The evolution of geographic variation in birdsong. Adv. Study Behav. 37, 403–458. (doi:10.1016/S0065-3454(07)37009-5)

[15] Krützen M, Willems EP, van Schaik CP 2011 Culture and geographic variation in orangutan behavior. Curr. Biol. 21, 1808–1812. (doi:10.1016/j.cub.2011.09.017) 22018539 10.1016/j.cub.2011.09.017

[16] Fayet AL, Tobias JA, Hintzen RE, Seddon N 2014 Immigration and dispersal are key determinants of cultural diversity in a songbird population. Behav. Ecol. 25, 744–753. (doi:10.1093/beheco/aru047)

[17] Riesch R, Barrett-Lennard LG, Ellis GM, Ford JKB, Deecke VB 2012 Cultural traditions and the evolution of reproductive isolation: ecological speciation in killer whales? Biol. J. Linn. Soc. 106, 1–17. (doi:10.1111/j.1095-8312.2012.01872.x)

[18] Nunn CL, Thrall PH, Bartz K, Dasgupta T, Boesch C 2009 Do transmission mechanisms or social systems drive cultural dynamics in socially structured populations? Anim. Behav. 77, 1515–1524. (doi:10.1016/j.anebehav.2009.02.023)

[19] Cantor M, Shoemaker LG, Cabral RB, Flores CO, Varga M, Whitehead H 2015 Multilevel animal societies can emerge from cultural transmission. Nat. Commun. 6, 8091 (doi:10.1038/ncomms9091) 26348688 10.1038/ncomms9091 PMC4569709

[20] Janik VM, Slater PJB 1997 Vocal learning in mammals. Adv. Study Behav. 26, 59–99. (doi:10.1016/S0065-3454(08)60377-0)

[21] Janik V, Slater P 2000 The different roles of social learning in vocal communication. Anim. Behav. 60, 1–11. (doi:10.1006/anbe.2000.1410) 10924198 10.1006/anbe.2000.1410

[22] Janik VM 2014 Cetacean vocal learning and communication. Curr. Opin. Neurobiol. 28C, 60–65. (doi:10.1016/j.conb.2014.06.010) 25057816 10.1016/j.conb.2014.06.010

[23] Rendell L, Whitehead H 2003 Vocal clans in sperm whales (*Physeter macrocephalus*). Proc. R. Soc. Lond. B 270, 225–231. (doi:10.1098/rspb.2002.2239) 10.1098/rspb.2002.2239 PMC1691237 12614570

[24] Gero S, Whitehead H, Rendell L 2016 Individual, unit and vocal clan level identity cues in sperm whale codas. R. Soc. open sci. 3, 150372 (doi:10.1098/rsos.150372) 26909165 10.1098/rsos.150372 PMC4736920

[25] Watkins WA, Schevill WE 1977 Sperm whale codas. J. Acoust. Soc. Am. 62, 1486–1490. (doi:10.1121/1.381678)

[26] Rendell L, Mesnick SL, Dalebout ML, Burtenshaw J, Whitehead H 2012 Can genetic differences explain vocal dialect variation in sperm whales, *Physeter macrocephalus*? Behav. Genet. 42, 332–343. (doi:10.1007/s10519-011-9513-y) 22015469 10.1007/s10519-011-9513-y

[27] Boyd R, Richerson PJ 1985 Culture and the evolutionary process. Chicago, IL: Macmillan Publishers Limited.

[28] McPherson M, Smith-Lovin L, Cook JM 2001 Birds of a feather: homophily in social networks. Annu. Rev. Sociol. 27, 415–444. (doi:10.1146/annurev.soc.27.1.415)

[29] Antunes R 2009 Variation in sperm whale (*Physeter macrocephalus*) coda vocalization and social structure in the North Atlantic Ocean. PhD thesis, University of St Andrews, St Andrews, UK.

[30] Whitehead H, Rendell L 2004 Movements, habitat use and feeding success of cultural clans of South Pacific sperm whales. J. Anim. Ecol. 73, 190–196. (doi:10.1111/j.1365-2656.2004.00798.x)

[31] Gero S et al. 2014 Behavior and social structure of the sperm whales of Dominica, West Indies. Mar. Mamm. Sci. 30, 905–922. (doi:10.1111/mms.12086)

[32] Gero S, Gordon JCD, Whitehead H 2015 Individualized social preferences and long-term social fidelity between social units of sperm whales. Anim. Behav. 102, 15–23. (doi:10.1016/j.anbehav.2015.01.008)

[33] Gero S, Gordon JCD, Whitehead H 2013 Calves as social hubs: dynamics of the social network within sperm whale units. Proc. R. Soc. B 280, 20131113 (doi:10.1098/rspb.2013.1113) 10.1098/rspb.2013.1113 PMC3774244 23740785

[34] Gero S, Engelhaupt D, Whitehead H 2008 Heterogeneous social associations within a sperm whale, *Physeter macrocephalus*, unit reflect pairwise relatedness. Behav. Ecol. Sociobiol. 63, 143–151. (doi:10.1007/s00265-008-0645-x)

[35] Gero S, Gordon JCD, Carlson C, Evans P, Whitehead H 2007 Population estimate and inter-island movement of sperm whales, *Physeter macrocephalus*, in the Eastern Caribbean. J. Cetacean Res. Manag. 9, 143–150.

[36] Gero S, Engelhaupt D, Rendell L, Whitehead H 2009 Who cares? Between-group variation in alloparental caregiving in sperm whales. Behav. Ecol. 20, 838–843. (doi:10.1093/beheco/arp068)

[37] Gero S, Whitehead H 2007 Suckling behavior in sperm whale calves: observation and hypotheses. Mar. Mamm. Sci. 23, 398–413. (doi:10.1111/j.1748-7692.2007.00113.x)

[38] Antunes R, Schulz T, Gero S, Whitehead H, Gordon JCD, Rendell L 2011 Individually distinctive acoustic features in sperm whale codas. Anim. Behav. 81, 723–730. (doi:10.1016/j.anbehav.2010.12.019)

[39] Schulz TM, Whitehead H, Gero S, Rendell L 2011 Individual vocal production in a sperm whale (*Physeter macrocephalus*) social unit. Mar. Mamm. Sci. 27, 149–166. (doi:10.1111/j.1748-7692.2010.00399.x)

[40] Schulz TM, Whitehead H, Gero S, Rendell L 2008 Overlapping and matching of codas in vocal interactions between sperm whales: insights into communication function. Anim. Behav. 76, 1977–1988. (doi:10.1016/j.anbehav.2008.07.032)

[41] Rendell L, Whitehead H 2003 Comparing repertoires of sperm whale codas: a multiple methods approach. Bioacoustics 14, 61–81. (doi:10.1080/09524622.2003.9753513)

[42] Ankerst M, Breunig MM, Kriegel H-P, Sander J 1999 OPTICS: ordering points to identify the clustering structure. ACM SIGMOD Rec. 28, 49–60. (doi:10.1145/304181.304187)

[43] Achtert E, Kriegel H-P, Schubert E, Zimek A 2013 *Interactive data mining with 3D-parallel-coordinate-trees*. In *Proc. 2013 ACM SIGMOD Int. Conf. Manag. Data, New York, NY, 22–27 June*, pp. 1009–1012. New York, NY: ACM. (doi:10.1145/2463676.2463696)

[44] Weilgart L, Whitehead H 1997 Group-specific dialects and geographical variation in coda repertoire in South Pacific sperm whales. Behav. Ecol. Sociobiol. 40, 277–285. (doi:10.1007/s002650050343)

[45] Mantel N 1967 The detection of disease clustering and generalized regression approach. Cancer Res. 27, 209–220. 6018555

[46] Schnell GD, Watt DJ, Douglas ME 1985 Statistical comparison of proximity matrices: applications in animal behaviour. Anim. Behav. 33, 239–253. (doi:10.1016/S0003-3472(85)80138-X)

[47] Whitehead H 2009 SOCPROG programs: analysing animal social structures. Behav. Ecol. Sociobiol. 63, 765–778. (doi:10.1007/s00265-008-0697-y)

[48] Bridge PD 1993 *Classification* In Biological data analysis (ed. Fry JC), pp. 219–242. Oxford, UK: Oxford University Press.

[49] Cairns SJ, Schwager SJ 1987 A comparison of association indices. Anim. Behav. 35, 1454–1469. (doi:10.1016/S0003-3472(87)80018-0)

[50] Engelhaupt D et al. 2009 Female philopatry in coastal basins and male dispersion across the North Atlantic in a highly mobile marine species, the sperm whale (*Physeter macrocephalus*). Mol. Ecol. 18, 4193–4205. (doi:10.1111/j.1365-294X.2009.04355.x) 19769692 10.1111/j.1365-294X.2009.04355.x

[51] Torres AMA 2006 Distincion de clanes de cachalote (*Physeter macrocephalus*) en el Golfo de California, mediante comparacion de repertorios de codas. Master's thesis, CICIMAR, Instituto Politécnico Nacional, Mexico.

[52] Whitehead H, Coakes AK, Jaquet N, Lusseau S 2008 Movements of sperm whales in the tropical Pacific. Mar. Ecol. Prog. Ser. 361, 291–300. (doi:10.3354/meps07412)

[53] Whitehead H, Antunes R, Gero S, Wong SNP, Engelhaupt D, Rendell L 2012 Multilevel societies of female sperm whales (*Physeter macrocephalus*) in the Atlantic and Pacific: Why are they so different? Int. J. Primatol. 33, 1142–1164. (doi:10.1007/s10764-012-9598-z)

[54] Kirschel ANG, Blumstein DT, Smith TB 2009 Character displacement of song and morphology in African tinkerbirds. Proc. Natl Acad. Sci. USA 106, 8256–8261. (doi:10.1073/pnas.0810124106) 19420223 10.1073/pnas.0810124106 PMC2688847

[55] Whitehead H, Gero S 2015 Conflicting rates of increase in the sperm whale population of the eastern Caribbean: positive observed rates do not reflect a healthy population. Endanger. Species Res. 27, 207–218. (doi:10.3354/esr00657)

[56] Delibes M, Gaona P, Ferreras P 2001 Effects of an attractive sink leading into maladaptive habitat selection. Am. Nat. 158, 277–285. (doi:10.1086/321319) 18707324 10.1086/321319

[57] Battin J 2004 When good animals love bad habitats: ecological traps and the conservation of animal populations. Conserv. Biol. 18, 1482–1491. (doi:10.1111/j.1523-1739.2004.00417.x)

[58] Catchpole CK, Slater PBJ 2013 Bird song: biological themes and variations. Cambridge, UK: Cambridge University Press.

[59] Efferson C, Lalive R, Fehr E 2008 The coevolution of cultural groups and ingroup favoritism. Science 321, 1844–1849. (doi:10.1126/science.1155805) 18818361 10.1126/science.1155805

[60] Marcoux M, Whitehead H, Rendell L 2007 Sperm whale feeding variation by location, year, social group and clan: evidence from stable isotopes. Mar. Ecol. Prog. Ser. 333, 309–314. (doi:10.3354/meps333309)

[61] Marcoux M, Rendell L, Whitehead H 2007 Indications of fitness differences among vocal clans of sperm whales. Behav. Ecol. Sociobiol. 61, 1093–1098. (doi:10.1007/s00265-006-0342-6)

[62] Cantor M, Whitehead H 2015 How does social behavior differ among sperm whale clans? Mar. Mamm. Sci. 31, 1275–1290. (doi:10.1111/mms.12218)

[63] Tønnessen JN, Johnsen AO 1982 The history of modern whaling. Berkeley, CA: University of California Press.

