# Supplementary material for: Socially segregated, sympatric sperm whale clans in the Atlantic Ocean
Source: R Soc Open Sci. 2016 Jun 8;3(6):160061. doi: 10.1098/rsos.160061 (PMC4929901; doi:10.1098/rsos.160061)
Supplement: Gero et al - FULL ESM. This document contains description of supplementary methodological details, as well as supporting figures and tables. [file rsos160061supp1.docx]

Supplementary Material for Gero *et al.* (XXXX) Socially Segregated, Sympatric Sperm Whale Clans in the Atlantic Ocean. RSOS.

**Field Effort:**

Field work totaled 3660 hours with whales on 402 days across 472 days of effort from 2005-2015 on one of four platforms: a dedicated 12m auxiliary sailing vessel, a dedicated 5m outboard skiff, a dedicated 11m outboard rigid-hull inflatable (RHIB) or an 18m whale-watch vessel. Effort is broken down by year and platform in Table S1.

Table S1: Effort across years

| **Year** | **Start Date** | **End Date** | **Days Effort** | **Platform** |
| --- | --- | --- | --- | --- |
| **2005** | January 14 | April 13 | 62 | Sailing only |
| **2006** | January 17 | February 11 | 21 | Whalewatch only |
| **2007** | January 28 | February 28 | 30 | Skiff and Whalewatch |
| **2008** | February 8 | May 8 | 75 | All |
| **2009** | January 11 | March 29 | 64 | Skiff and Whalewatch |
| **2010** | January 20 | April 18 | 72 | Sailing only |
| **2011** | March 3 | April 12 | 35 | RHIB only |
| **2012** | May 5 | June 6 | 31 | Sailing only |
| **2013** |  |  | 0 | NO EFFORT |
| **2014** | April 2 | May 12 | 26 | RHIB only |
| **2015** | February 11 | April 12 | 56 | RHIB and Sailing |

Recording Systems:

Vocalizations were recorded using one of several recording setups: In 2005, we used a Fostex VF-160 multitrack recorder (44.1 kHz sampling rate) and a custom built towed hydrophone (Benthos AQ-4 elements, frequency response: 0.1-30kHz) with a filter box with high-pass filters up to 1 kHz resulting in a recording chain with a flat frequency response across a minimum of 2 - 20kHz. No recordings were made during the short 2006 season. In the 2007, 2009, and 2011 seasons, we used a Zoom H4 portable field recorder (48 kHz sampling rate) and a Cetacean Research Technology C55 hydrophone (frequency response: 0.02-44kHz) and no filters. During the 2008, 2010, 2012, and 2015 seasons, we used the custom-built towed hydrophone (Benthos AQ-4 elements, frequency response: 0.1-30kHz) with a filter box with high-pass filters up to 1 kHz resulting in a recording chain with a flat frequency response across a minimum of 2 - 20 kHz. This was connected to a computer based recording system as a part of the International Fund for Animal Welfare’s (IFAW) LOGGER software package (48 kHz sampling rate) or PAMGUARD (minimum 48 kHz sampling rate). The variation in the frequency responses and sampling rates of the recording systems used did not affect our ability to record clean signals for both the coda and echolocation clicks produced by sperm whales; and as a result, the temporal patterning of clicks used in this analysis.

Measuring Inter-click Interval methodological detail:

Coda recordings were then analyzed using Rainbow Click software to determine the inter-click intervals (ICI, the time between the onset of one click and the onset of the next click) defining the temporal structure of all codas recorded. The codas were marked manually by a two trained observers (SG analyzed all recordings for the 9 units in the Eastern Caribbean Clan, and AB analyzed recordings for units P and K after which they were evaluated by SG for consistency) and the timing of the clicks within codas calculated by the software. Each coda could then be represented by the set of ICIs, using the absolute length of the intervals given previous research suggested that more information may be encoded in the absolute than when standardizing relative to total coda length [15]. Rare long coda types (>10 clicks; <5% of all codas recorded) were excluded from the analysis.

### Classification-free Similarity of Repertoires

We calculated the similarity between the coda repertoires of two units, *A* and *B*, in the same way as previous studies [1–3]:

where *S_AB_* is the similarity between repertoires *A* and *B* each with *n_A_* and *n_B_* codas, respectively; *l_i_* and *l_j_* are the number of clicks in coda *i* from repertoire *A* and the number of clicks in coda *j* in repertoire *B*; *b* is the basal similarity [here set to 0.001 seconds, which gives a very fine scale comparison of codas on the order of 1 ms, but the results are robust to variation in b, see 2,4] and *d_ij_* is the Euclidean distance between the ICI vectors of codas *i* and *j*. The equation implicitly assigns multivariate similarity between two codas containing different numbers of clicks to zero. Similarities were calculated using custom-written routines in *MATLAB* 7.12 (The Mathworks, Inc., MA, USA).


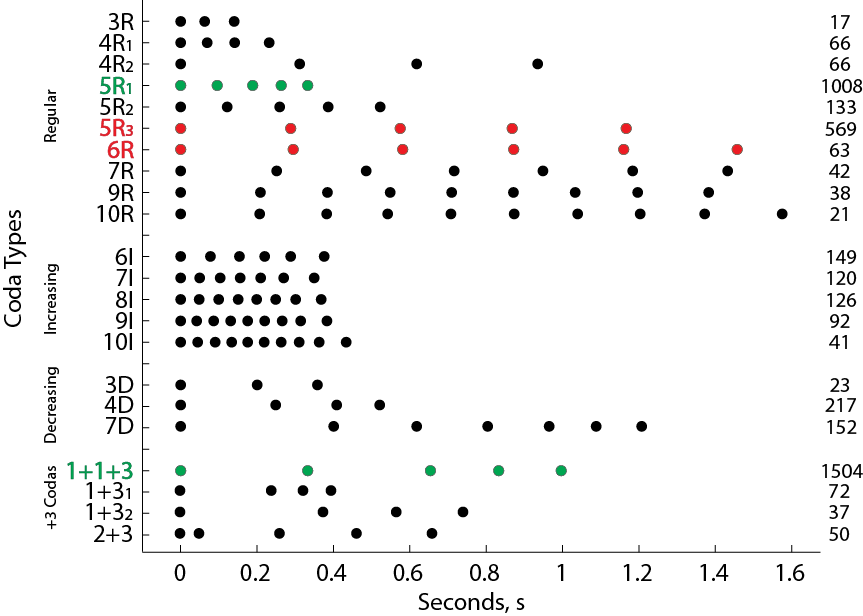
Rhythm plot for all coda types

Figure S1 - Rhythm plot illustrating the mean timing of the clicks in all 22 coda types. Types shaded in green are red are the predominant coda types produced by the two clans. The sample sizes for each coda type are given on the right axis.

Sample Sizes and Discovery Curves for Unit Repertoires

While discovery curves of the coda types are not absolutely asymptotic, they suggest that the coda type repertoire of a given unit is adequately sampled once approximately 300 codas have been recorded; at which point only a few rare coda types remain. While the two units from the EC2 clan have only been sampled on a few days, we have collected very near the average sample of 418 codas (range 247-1099 codas; omitting the large sample for Unit F the mean is 350 codas; Table S2). We feel therefore that we have adequately sampled the potential different coda types produced by all units. Furthermore, the key differences between clans (the different prevalent codas used between the two sets of units) would be apparent even with smaller samples. For example, the EC1 clan produces at least one 1+1+3 coda in 59.3% of all recordings, which it was only recorded in 7.1% of recordings from EC2 Clan; and vice versa we recorded the 5R3 in 92.6% of recordings of the EC2 clan and only in 2.1% of the EC1 clan’s recordings. In addition, we tested the robustness of the similarity results using 1000 bootstrap replicates in which the repertoires were sampled with replacement prior to recalculating the pairwise similarities. Strong bootstrap support dividing the two clans in 100% of the 1000 bootstrap replicates supports our conclusions and gives confidence that the division is not dependent on sampling.


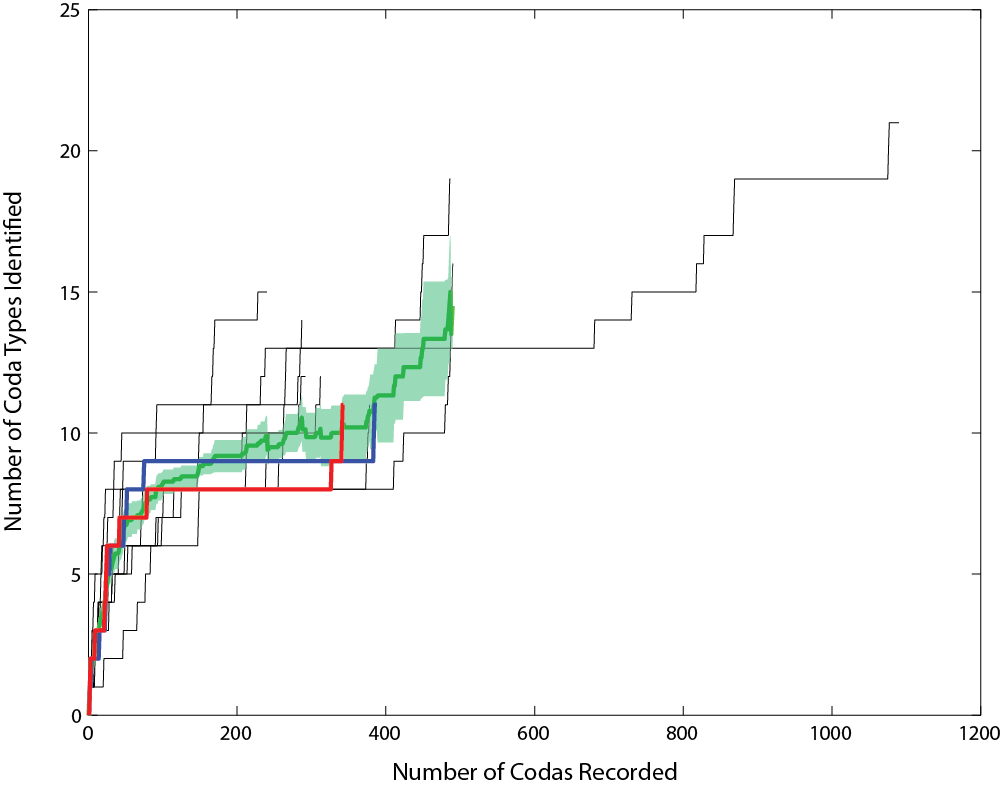


Figure S2 – Discovery curves of coda types for all units. Units P and K are labelled in red and blue, respectively. Mean for all units in green ± 1 SE (shaded green).


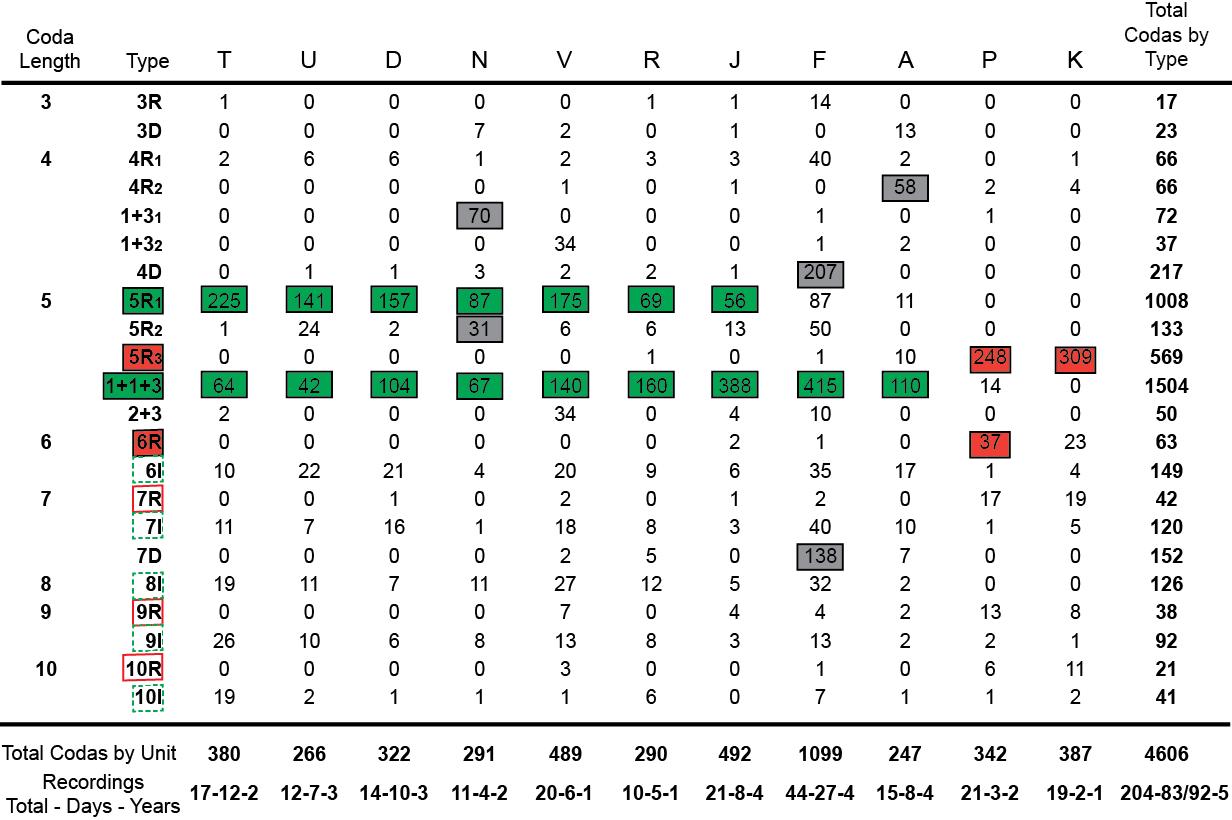


Coda Repertoires with Counts of Types

**Table S2 - Coda repertoires of 11 units of sperm whales recorded off Dominica compared based on OPTICS categorical classification of coda types. Letters denote units. Green fill denotes predominant codas (>10 % of total production of the unit) in the EC1 clan, while red fill marks predominant codas for the two rare units in the EC2 clan (P and K). Dashed boxes indicate difference between two clans in less common coda types as seen in PCA plots in Figure S3. Numbers below each column are the total number of codas recorded from each unit, as well as the total number of recordings, recording days, and years per unit. On 8 days recordings were made of different units and so that day was counted once as a recording day for each unit in the unit totals, therefore there is a difference in the two totals for days (Unique Calendar Days/Unit Days).**

Principal components plot of all codas 5 to 10 clicks long:

Principal components analysis was conducted on raw absolute ICI data as variables. The first principal component is related to total coda duration in seconds as all ICI loadings are positively related to PC1. PC2 generally reflects the tradeoff between the duration of the first versus last ICI (right column of plots in S3). Three and four click codas were omitted from this figure as the two rare units produced very few (8 codas in total across 7 types); and so do not serve to highlight differences between clans other than that evidently the rare units produce them infrequently.

There is a clear division between the codas made by the two rare units and those made by the nine common units for all coda lengths (right column of subplots in Figure S3). The predominant coda types of both clans are 5 or 6 clicks in length (subplot A and C in Figure S3).

Furthermore, there are also consistent differences between the non-predominant types produce by the two clans (coda lengths between 7 and 10 clicks). Note the repeated patterns of the short “I” increasing class codas on the left side of the plot (generally produced by the 9 common units) compared to the division between the “R” regular class codas (generally produced by the two rare units) on the left of the plots (see also the rhythm plot in figure S1 above).

Note that in some cases a number of codas were omitted as noise by the OPTICS algorithm. This is due to either not meeting the point density requirement to be designated a new cluster or for having too few points to be classified a new type. For example, the diffuse cluster in the bottom right of the 8 click coda subplot (G). These are mostly made by the two rare units and have a rhythm of a purported “8R” coda. However, these did not meet the density requirement set for the OPTICS algorithm. Similarly, the diffuse cluster in the upper right side of the 6 click subplot (C) which have the rhythm of a purported “6D” coda but which did not meet the density requirement in OPTICS. Nonetheless, the codas classified as noise were used in the classification-free similarity measure (see figure 1 in main text).


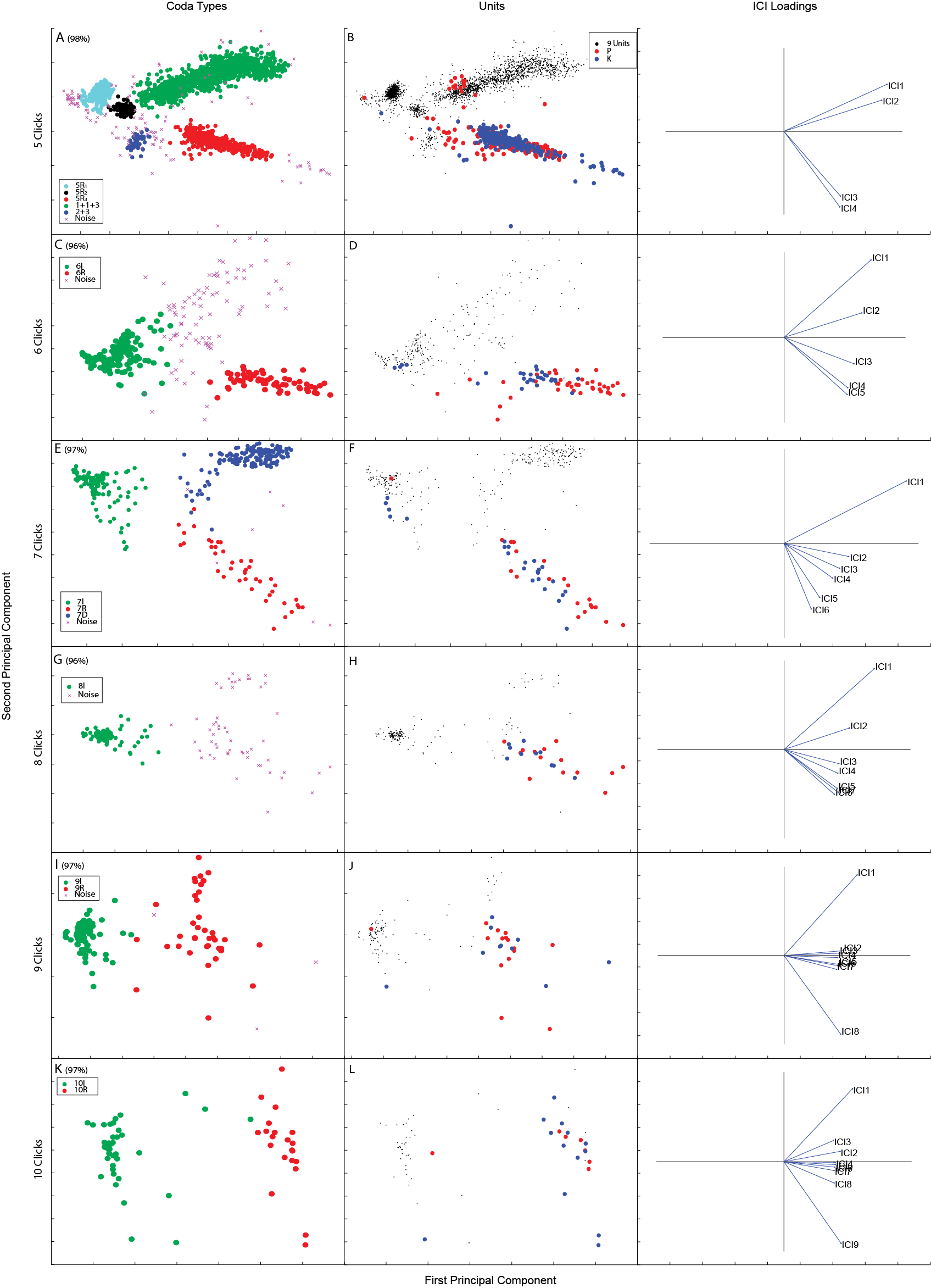


Figure S3 - First two principal components for all 5 to 10 click codas colored by coda type (left column) and unit (middle column) with ICI loadings (right column). PC1 relates positively to total coda duration in seconds as all ICI loadings are positively related. PC2 reflects the tradeoff between the duration of the first and last ICI in a coda. Percentage in parenthesis (left column) denotes the variance explained by the first two principal components.


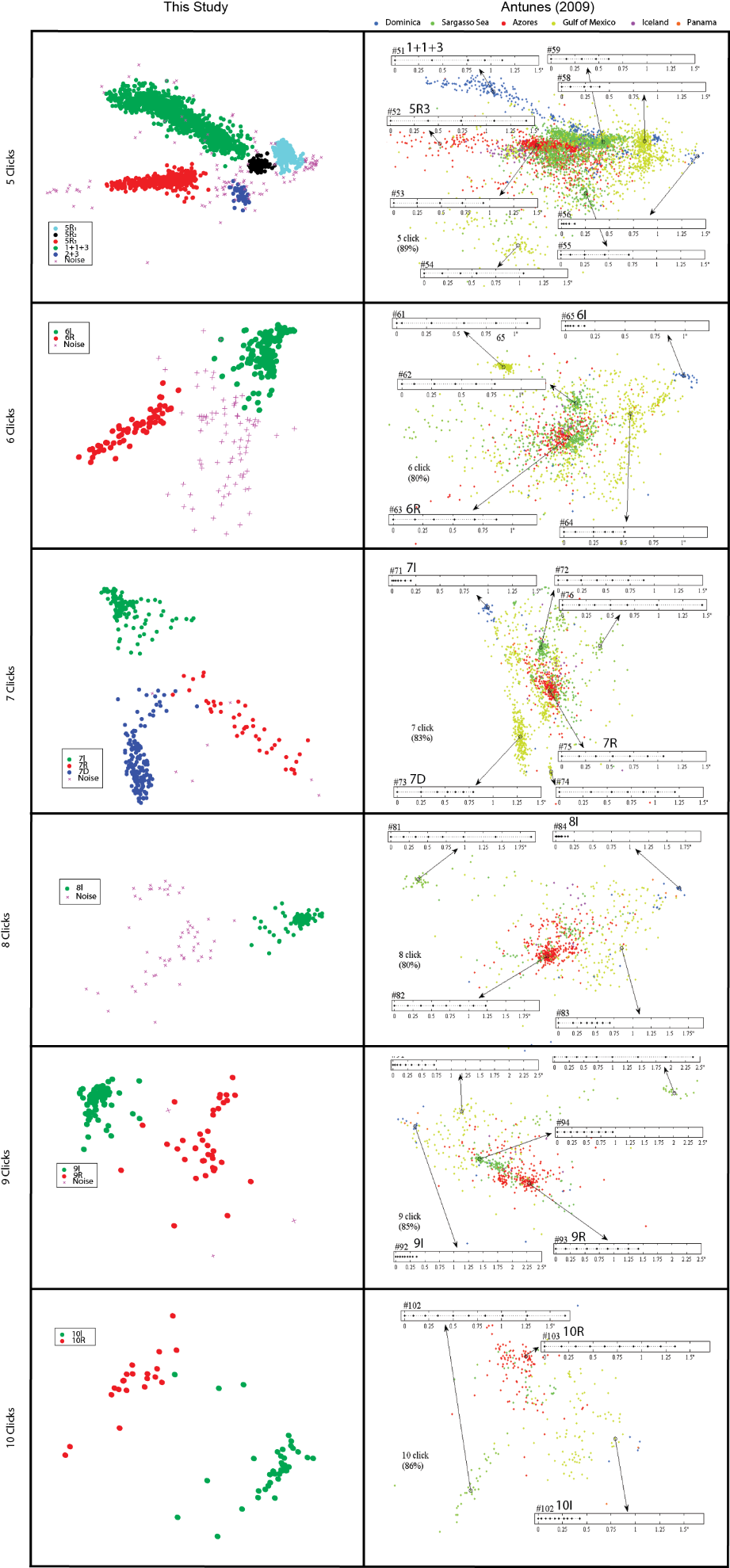
Caribbean codas contrasted with wider North Atlantic:

Figure S4 - Principal components plots from this study (left column) rotated to align with PCA plots from Antunes [5, reproduced from Figures 5.3 and 5.4 on pages 81 and 82, respectively] . Caribbean coda types labelled in Antunes plots by name. This suggests that the EC2 clan may originate from wider North Atlantic. Note that while rotated, these plots are not scaled to the same axes.

References:

1. Rendell, L. & Whitehead, H. 2003 Vocal clans in sperm whales (*Physeter macrocephalus*). *Proc. R. Soc. B.* **270**, 225–231. (doi:10.1098/rspb.2002.2239)

2. Rendell, L. & Whitehead, H. 2003 Comparing repertoires of sperm whale codas: A multiple methods approach. *Bioacoustics* **14**, 61–81.

3. Schulz, T. M., Whitehead, H., Gero, S. & Rendell, L. 2011 Individual vocal production in a sperm whale (*Physeter macrocephalus*) social unit. *Mar. Mammal Sci.* **27**, 149–166.

4. Gero, S. 2012 On the dynamics of social relationships and vocal communication between individuals and social units of sperm whales. **PhD thesis**. Department of Biology, Dalhousie University, Halifax, Canada. 146pp + xvii

5. Antunes, R. 2009 Variation in sperm whale (*Physeter macrocephalus*) coda vocalization and social structure in the North Atlantic Ocean. **Ph.D. Biol.** School of Biology, Univesity of St. Andrews, St. Andrews, Scotland, UK. 123pp. + xi
